# Editorial: Prevalence and transmission of emerging and replicating animal viruses

**DOI:** 10.3389/fcimb.2025.1683873

**Published:** 2025-09-09

**Authors:** Mohammad Enamul Hoque Kayesh, Ning Kong, Li Liangliang, Yongkun Du, Rui M. Gil da Costa

**Affiliations:** ^1^ Department of Microbiology and Public Health, Faculty of Animal Science and Veterinary Medicine, Patuakhali Science and Technology University, Barishal, Bangladesh; ^2^ Transboundary Animal Diseases Center, Joint Faculty of Veterinary Medicine, Kagoshima University, Kagoshima, Japan; ^3^ Shanghai Veterinary Research Institute, Chinese Academy of Agricultural Sciences, Shanghai, China; ^4^ College of Agricultural Science and Engineering, Liaocheng University, Liaocheng, China; ^5^ Henan Agricultural University, Zhengzhou, China; ^6^ Post-Graduate Program in Adult Health, Federal University of Maranhão, São Luís, Brazil; ^7^ Department of Pathology, State University of Maranhão, São Luís, Brazil

**Keywords:** emerging animal viruses, prevalence, transmission, bacteriophage, zoonotic disease

Emerging and re-emerging viral pathogens represent an increasingly significant threat to global public and animal health (Luo and Gao, 2020; Baker et al., 2022). Approximately 75% of all emerging infectious diseases are of zoonotic origin, with viruses accounting for a substantial proportion of it (Leal Filho et al., 2022; Sharan et al., 2023). The emergence and spread of viruses are shaped by complex evolutionary dynamics, including mutation, recombination, and host adaptation, which pose significant challenges to both veterinary and public health. These threats are further augmented by global climate change and increased international trade, which facilitate the transmission of viral pathogens across geographic and species boundaries. Addressing these risks requires sustained research into the prevalence and transmission patterns of emerging animal viruses across varied ecosystems. Together, these factors underscore the urgent need for integrated, evidence-based, and proactive approaches to detect, monitor, and control the spread of emerging and re-emerging animal viruses.

The Research Topic “Prevalence and Transmission of Emerging and Replicating Animal Viruses” brings together five recent studies that collectively advance our understanding of viral pathogenesis, detection and control. These investigations contribute to the development of more rapid diagnostic tools and explore alternative therapeutic strategies, offering valuable insights for the management of emerging viral infections in animal populations.

Phage therapy is increasingly recognized as a targeted approach within personalized medicine for both human and veterinary use, and has recently emerged as a promising strategy for controlling foodborne pathogens and enhancing food safety (Ferriol-Gonzalez and Domingo-Calap, 2021; Loponte et al., 2021). *Salmonella Abortusequi* (*S. Abortusequi*) is responsible for abortions in equine animals, and serious foodborne illness, which control is critical. Cao et al. demonstrated that the vB_SalP_LDDK01 phage could effectively destroy the biofilm of *S. Abortusequi* and also reduce the burden of *S. Abortusequi* from contaminated donkey meat. The overall findings of the study indicate that the vB_SalP_LDDK01 phage is a promising biological agent for the inhibition of *S. Abortusequi* in donkey meat.

Zhang et al. provide important insights into the prevalence and genetic characteristics of intestinal protozoa and microsporidia in domestic cats in Anhui Province, China. Although overall infection rates were low, the detection of zoonotic genotypes of *Giardia intestinalis*, *Cryptosporidium felis*, and *Enterocytozoon bieneusi* highlights the potential risk for human transmission. These findings highlight the importance of considering domestic cats as potential reservoirs for human infection in both clinical and public health contexts.

Currently, four types of porcine epidemic diarrhea viruses have been identified: swine acute diarrhea syndrome coronavirus (SADS-CoV), porcine epidemic diarrhea virus, porcine deltacoronavirus, and transmissible gastroenteritis virus (Liu and Wang, 2021). The clinical symptoms of SADS-CoV closely resemble those of the other three viruses, making differential diagnosis challenging in clinical settings, and there are no effective treatments or vaccines available for SADS-CoV to date (Liu and Wang, 2021). Therefore, establishing a rapid detection method for SADS-CoV is crucial for early detection, control, and prevention. Cong et al. introduces a highly sensitive and specific fluorescent microsphere-based immunochromatographic assay for point-of-care detection of SADS-CoV, demonstrating a 97.22% concordance with qPCR. The development of this robust point-of-care testing platform by Cong et al. represents a significant step forward in early intervention and containment strategies for this economically devastating disease.

The development of a rapid and cost-effective tool is also critically needed for efficient bovine herpesvirus type 1 (BHV-1) surveillance in the livestock industry. Liu et al. developed a rapid, cost-effective indirect ELISA based on recombinant gD protein for the detection of BHV-1 antibodies, and this assay demonstrated high specificity and strong agreement with commercial kits, offering a promising tool for large-scale epidemiological monitoring in cattle herds.

Liu et al. combine traditional Chinese medicine with modern analytical tools to develop and evaluate two optimized Maxing Shigan Decoction (MXSG) formulations for treating infectious bronchitis virus (IBV) in poultry. Through *in vitro* and *in vivo* validation, as well as LC-MS and network pharmacology, Liu et al. reveal multi-target mechanisms underlying the enhanced efficacy of MXSG-mix, particularly through acting on AKT Serine/Threonine Kinase 1 and Caspase 3 and downregulation of B-cell lymphoma 2 expression, providing insights into its anti-IBV mechanism. This integrated approach highlights a novel paradigm for advancing veterinary applications of traditional Chinese medicine.

These recent advancements underscore the critical role of innovative diagnostics and integrative therapeutics in veterinary virology. The development of rapid, field-compatible assays for SADS-CoV and BHV-1 enhances early detection and large-scale surveillance capabilities, essential for timely outbreak control. Simultaneously, the successful application of optimized traditional formulations against IBV highlights the potential of combining ethnoveterinary knowledge with modern science, paving the way for more holistic and sustainable approaches to managing viral infections in livestock.
